# Diminished COX-2/PGE_2_-Mediated Antiviral Response Due to Impaired NOX/MAPK Signaling in G6PD-Knockdown Lung Epithelial Cells

**DOI:** 10.1371/journal.pone.0153462

**Published:** 2016-04-20

**Authors:** Hsin-Ru Lin, Yi-Hsuan Wu, Wei-Chen Yen, Chuen-Mao Yang, Daniel Tsun-Yee Chiu

**Affiliations:** 1 Molecular Medicine Research Center, Chang Gung University, Taoyuan City, Taiwan; 2 Department of Medical Biotechnology and Laboratory Science, College of Medicine, Chang Gung University, Taoyuan City, Taiwan; 3 Graduate Institute of Biomedical Science, College of Medicine, Chang Gung University, Taoyuan City, Taiwan; 4 Healthy Aging Research Center, Chang Gung University, Taoyuan City, Taiwan; 5 Department of physiology and pharmacology, College of Medicine, Chang Gung University, Taoyuan City, Taiwan; 6 Department of Pediatric Hematology, Chang Gung Memorial Hospital, Lin-Kou, Taiwan; Chang Gung University of Science and Technology, TAIWAN

## Abstract

Glucose-6-phosphate dehydrogenase (G6PD) provides the reducing agent NADPH to meet the cellular needs for reductive biosynthesis and the maintenance of redox homeostasis. G6PD-deficient cells experience a high level of oxidative stress and an increased susceptibility to viral infections. Cyclooxygenase-2 (COX-2) is a key mediator in the regulation of viral replication and inflammatory response. In the current study, the role of G6PD on the inflammatory response was determined in both scramble control and G6PD-knockdown (G6PD-kd) A549 cells upon tumor necrosis factor-α (TNF-α) stimulation. A decreased expression pattern of induced COX-2 and reduced production of downstream PGE_2_ occurred upon TNF-α stimulation in G6PD-kd A549 cells compared with scramble control A549 cells. TNF-α-induced antiviral activity revealed that decreased COX-2 expression enhanced the susceptibility to coronavirus 229E infection in G6PD-kd A549 cells and was a result of the decreased phosphorylation levels of MAPK (p38 and ERK1/2) and NF-κB. The impaired inflammatory response in G6PD-kd A549 cells was found to be mediated through NADPH oxidase (NOX) signaling as elucidated by cell pretreatment with a NOX2-siRNA or NOX inhibitor, diphenyleneiodonium chloride (DPI). In addition, NOX activity with TNF-α treatment in G6PD-kd A549 cells was not up-regulated and was coupled with a decrease in NOX subunit expression at the transcriptional level, implying that TNF-α-mediated NOX signaling requires the participation of G6PD. Together, these data suggest that G6PD deficiency affects the cellular inflammatory response and the decreased TNF-α-mediated antiviral response in G6PD-kd A549 cells is a result of dysregulated NOX/MAPK/NF-κB/COX-2 signaling.

## Introduction

Glucose-6-phosphate dehydrogenase (G6PD), the rate-limiting enzyme of the pentose monophosphate shunt, is ubiquitously expressed in human tissues [1]. G6PD involves the oxidation of glucose-6-phosphate to 6-phosphogluconolactone, which then produces a reduced form of nicotinamide adenine dinucleotide phosphate (NADPH) to fulfill the cellular needs for cellular reductive biosynthesis and redox balance [2]. G6PD deficiency affects cellular functions in nucleated cells, including dysregulated cellular signaling, increased cell senescence or apoptosis and enhanced susceptibility to viral infection [1]. G6PD deficiency increases the risk for degenerative diseases [3–6]. Knockdown of G6PD by RNA interference renders HepG2 cells highly susceptible to H_2_O_2_-induced cell death because of impaired dephosphorylation signaling [7]. In macrophages, G6PD increases the activation of the p38 MAPK (Mitogen-activated protein kinases) and NF-κB (Nuclear factor of kappa light polypeptide gene enhancer in B-cells) pathways, which may lead to an increased inflammatory response [8]. These findings indicate that the G6PD plays an important role in modulating cellular signaling and physiological responses.

Airway epithelial cells are the first barrier of defense in the lung and are equipped with multiple lines of innate defense mechanisms to fight against invading pathogens, including viruses [9, 10]. Virus-infected airway epithelial cells express various cytokines that attract immune cells to combat infection and tissue damage [10]. Tumor necrosis factor-α (TNF-α) is a pleiotropic cytokine that plays an important role in orchestrating the immune response. It is induced in activated monocyte/macrophages, where its systemic effect promotes a network of inflammatory gene expression, including cytokines, adhesion molecules, and growth factors [11, 12].

The redox status influences the micro-environment in cells, which in turn affects physiological functions [13]. NADPH oxidases (NOXs) are a ROS (Reactive oxygen species) source in cells besides mitochondria [14–16]. NOXs are a family of proteins, including NOX1, NOX2, NOX3, NOX4, NOX5, Duox1 and Duox2, and play a major role in regulating cellular functions, especially membrane-bound NOX in different cell types [17, 18]. NOX uses NADPH as a substrate to produce ROS that can induce cell signaling or directly interact with pathogens to protect cells from infection [19–21]. A change in redox status has been implicated in initiating inflammatory responses through the activation of transcription factors, such as NF-κB, AP-1, and other signal transduction pathways, including MAPKs, leading to the enhanced expression of pro-inflammatory genes [22–24]. The inflammatory response is also a powerful weapon for the host to be able to fight against pathogen infections. Because G6PD plays a pivotal role in maintaining cellular redox homeostasis, it will be of paramount importance to delineate how G6PD deficiency can affect immune responses as a result of redox imbalance.

In the present study, TNF-α was used as a stimulus to elucidate whether G6PD knockdown affects the inflammatory response against viral infection. We characterized the effects and mechanisms of G6PD knockdown in the inflammatory response, and compared the antiviral response in scramble and G6PD-knockdown (G6PD-kd) A549 cells modulated by COX-2. The effects of G6PD knockdown on the induction level of COX-2 and PGE_2_ was analyzed. Furthermore, the involvement of signaling pathway known to mediate regulation of COX-2/PGE_2_ on coronavirus infection was identified. The results clearly show that the down-regulation of COX-2 and subsequent decline of PGE_2_ impair antiviral response in G6PD-kd A549 cells upon TNF-α treatment. Most importantly, we have provided evidence that G6PD plays an important role in activating NOX/MAPK/NF-κB/COX-2 cascade and protects cells against viral infection.

## Materials and Methods

### Reagents

We purchased Dulbecco's modified Eagle's medium (DMEM) from Invitrogen (Carlsbad, CA, USA). Fetal bovine serum (FBS) was obtained from Corning (Now York, USA). Recombinant human TNF-α was purchased from Peprotech (Hamburg, Germany). Diphenyleneiodonium chloride (DPI), U0126, SB203580, Celecoxib, Tanshinone ΙΙA, Lucigenin, NADPH and β-Actin (AC-15) antibodies were obtained from Sigma (St. Louis, MO, USA). Helenalin was acquired from Biomol (Plymouth Meeting, PA, USA). Anti-G6PD was obtained from Genesis Biotech (Taipei, Taiwan). Anti-TNFR1 (sc-52739), anti-COX-2 (sc-19999), anti-NOX-1 (sc-25545), anti-NOX2 (sc-20782), anti-phospho-c-JUN (sc-822), anti-p67^phox^ (sc-15342), anti-Rac1 (sc-217), donkey-anti-goat IgG-HRP (sc-2056), goat anti-rabbit IgG-HRP (sc-2004) and goat anti-mouse IgG-HRP (sc-2005) antibodies were obtained from Santa Cruz (Santa Cruz, CA, USA). We purchased anti-phospho-p38 MAPK (#9211), anti-phospho-ERK1/2 MAPK (#4377), anti-phospho IκBα (#2859) and anti-phospho p65 (#3031) antibodies from Cell Signaling (Danvers, MA, USA). Luciferase assay kit was purchased from Promega (Madison, WI, USA).

### Cell cultures and TNF-α treatment

Human lung adenocarcinoma A549 cell line was obtained from the American Type Culture Collection (Rockville, MD, USA) and cultured in DMEM medium supplemented with 10% FBS, 100 units/ml penicillin, and 100 μg/ml streptomycin (Gibco, USA) at 37°C and 5% CO_2_. G6PD-kd and scramble control A549 cells were established as previously described [25] by transfecting with either G6PD-RNAi or scrambled vector using LF2000 according to manufacturer's instructions (Invitrogen). Finally, the stably transfected cell lines were selected with 300 μg/ml G418, and the knockdown efficiency was verified using G6PD activity and western blot.

After seeding A549 cells for 24 h, the complete medium was removed and replaced by serum free DMEM medium for 24 h; TNF-α was treated and incubated for the indicated time intervals.

### G6PD activity assay

The activity of G6PD in A549 cells was determined by a method modified from previously described [25]. Briefly, cells were collected by centrifugation at 1,500 rpm for 5 min at 4°C and resuspended in lysis buffer (1% Triton X-100, 0.05% SDS, 150 mM NaCl, 50 mM Tris-HCl pH 7.4, 1 mM NaF and 1 mM EGTA). The cell suspension was disrupted by a vigorous vortex. The resulting lysate was cleared by centrifugation at 12, 000 rpm for 15 min at 4°C, and the supernatant was used in the assay. The mixture consisted of 25 μg of protein in 1 ml of assay buffer (4 mM NADP^+^, 50 mM MgCl_2_, 50 mM Tris–HCl (pH 8.0) and 4 mM glucose 6-phosphate). The Bradford method was used to determine protein concentration.

### Quantitative PCR analysis

Total RNA was extracted from A549 cells by using Trizol reagent (Invitrogen). cDNA was synthesized by using oligo-dT as the primer in the presence of reverse transcriptase (Superscript III, Invitrogen). The mRNA level of targeted gene was assayed by using SYBR Green reagents (Yeastern Biotech, Taipei, Taiwan) in a real time thermocycler (Bio-Rad, Taipei, Taiwan). The Results were based on at least three independent experiments, and the mean fold changes were calculated. β-Actin was used as an endogenous control for normalization. The target mRNA levels were examined using the following primer sets: *NOX2*, 5'-GCTATGAGGTGGTGATGTTAGT-3' (Forward) and 5'-CTTCAGATTGGTGGCGTTATTG-3' (Reverse); *NOX1*, 5'- GCAAATGCTGTCACCGATATTC-3' (Forward) and 5'- TGCAGATTACCGTCCTTATTCC-3' (Reverse); *Rac1*, 5'- CCTGATGCAGGCCATCAAG-3' (Forward) and 5'-AGTAGGGATATATTCTCCAGGAAATGC-3' (Reverse); *p67*^*phox*^, 5'-CGGACAAGAAGGACTGGAAG-3' (Forward) and 5'-ACATGCAGCCAATGTTGAAG-3' (Reverse); *β-actin*, 5'- TCCACCTTCCAGCAGATG-3' (Forward) and 5'- GTGTAACGCAACTAAGTCATAG-3' (Reverse).

### Human coronavirus 229E infection

The human coronavirus strain 229E was obtained from Dr. Lai MM (Academia Sinica, Taiwan) and reproduced as previously described [26]. Virus pools were stored at -70°C until used. Approximately 3 × 10^5^ cells were seeded in a 6–well culture plates. Until the time point after TNF-α treatment, the culture was subjected to a human coronavirus 229E infection. A549 cells were infected with human coronavirus 229E at the MOI of 0.1 PFU.

### Plaque Assay

A549 cells were infected with HCoV-229E (0.1 MOI) for 24 h. After infection, the viral titer was calculated according to the plaque formation on the A549 cells, as described previously [26].

### Measurement of PGE_2_ secretion

A549 cells were seeded in 6–well culture plates. After reaching confluence, the cells were treated with TNF-α (15 ng/ml) at 37°C. After treatment for the indicated time intervals, the culture medium was obtained and stored at − 80°C until examined. PGE_2_ enzyme immunoassay kit (Cayman, MI, USA) was used to analyze the secretion of PGE_2_ according to the manufacturer's instructions.

### Measurement of NOX activity

After treatment with TNF-α, cells were collected and centrifuged at 1,500 rpm for 12 min at 4°C. The cell pellet was resuspended in 1 × PBS, and kept on ice. NOX activity was measured by a method modified from a previously described method [27]. Briefly, the assay mixtures containing either lucigenin (20 μM) or NADPH (1 μM), and cell suspension (5 × 10^3^ cells) was added to initiate the reaction in a multi-mode microplate reader (Hidex, Turku, Finland). Chemiluminescence was continuously measured for 15 min, and the measurement of the protein concentration was used as a normalization strategy.

### Measurement of COX-2 promoter activity

The human COX-2 promoter activity was measured as described previously [28] by using Dual Luciferase Assay (Promega, Madison, WI, USA) with a GLOMAX luminometer. The cellular extract was assayed for luciferase activity normalized to Renilla luciferase levels. Data were presented relative to pGL3-basic levels (RLU).

### Transfection assay

Human siRNA of universal negative control (NC) and NOX2 were purchased from Sigma (St. Louis, USA). Transfection of target siRNAs (100 nM) was performed by using Lipofectamine 2000 reagent based on the manufacturer's instructions.

### Western blot analysis

After incubation, cells were washed with 1× PBS and lysed in sample buffer (5% SDS, 12.5% β-mercaptoethanol, 0.5 M Tris-HCl (pH 6.8) and 25% Glycerol). The prepared samples were resolved on a 12% SDS-PAGE, electro-transferred onto a PVDF membrane (Millipore, Billerica, MA, USA), and reacted with various antibodies for 24 h. The target proteins were detected with horseradish peroxidase-conjugated secondary antibodies for 1 h and visualized using the enhanced chemiluminescence substrate (PerkinElmer, Waltham, MA, USA) on Fuji SuperFilms.

### Statistical analysis

Results were represented as the mean ±SD from at least three independent experiments. The two-tailed Student’s *t* test was applied to investigate the difference between groups. A p value of ≤ 0.05 and ≤ 0.01 was considered statistically significant.

## Results

### G6PD knockdown diminishes the replication level of coronavirus through regulation of COX-2/PGE_2_ upon TNF-α stimulation

**Fig 1:**
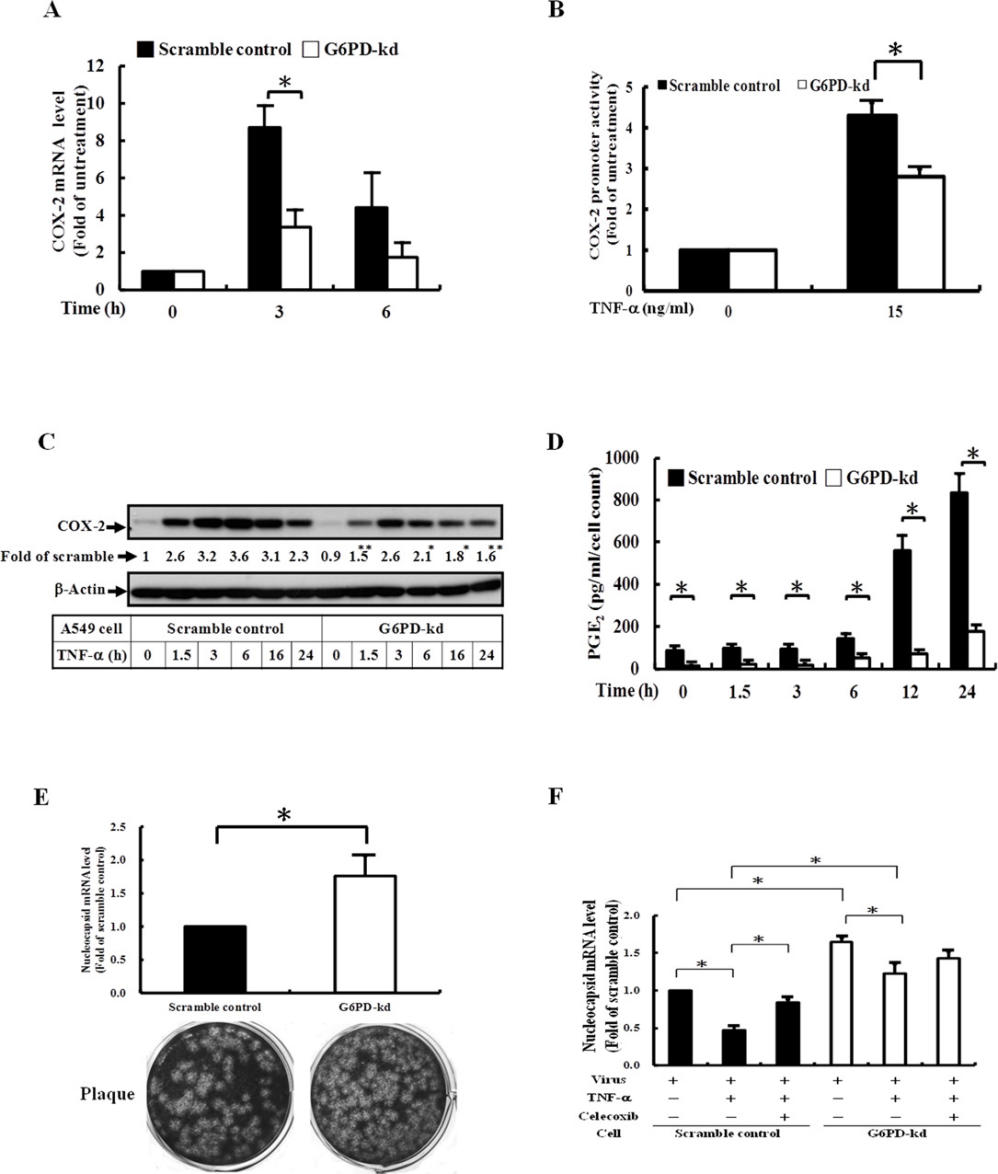
G6PD deficiency increases the replication level of coronavirus via down-regulation of TNF-α-induced COX-2 expression and its downstream metabolite PGE_2_ production in A549 cells. (A) Scramble control and G6PD-kd A549 were treated with 15 ng/ml TNF-α for the indicated time, and the expression of COX-2 mRNA was investigated by quantitative PCR. Data are reported as the means ±SD, n = 3. *p<0.05. (B) Scramble control and G6PD-kd A549 were treated with 15 ng/ml TNF-α for 24 h. COX-2 promoter activity was determined by the luciferase assay. Data are reported as the means ±SD, n = 3. *p<0.05. (C) The expression level of COX-2 protein upon 15 ng/ml TNF-α treatment at different time courses was shown, and β-actin was present as the loading control. Numbers represent relative fold differences of protein levels on the basis of densitometer quantitation. Data are means ±SD of three separate experiments, *p<0.05 and **p<0.01 represent levels of significant difference when comparing scramble control with TNF-α treatment at the corresponding time points. (D) PGE_2_ secretion by 15 ng/ml TNF-α stimulation was detected by ELISA. Data are reported as the means ±SD, n = 3. *p<0.05. (E) Upper panel: Scramble control and G6PD-kd A549 cells were infected with coronavirus (0.1 MOI) for 8 h, and the infected cells were harvested for analyzing viral mRNA expression. Data are reported as the fold change normalized to infected scramble control cells. Data are reported as the means ±SD, n = 3. *p<0.05. Lower panel: Scramble control and G6PD-kd A549 cells were infected with HCoV-229E (0.1 MOI) for 24 h then viral particle was harvested and virus titer was determined using plaque assay. (F) Scramble control and G6PD-kd A549 cells were infected with coronavirus (0.1 MOI) for 8 h upon 15 ng/ml TNF-α with or without 10 μM celecoxib co-pretreatment, and the infected cells were harvested for analyzing viral mRNA expression. Data are reported as the fold normalized to infected control cells. Data are reported as the means ±SD, n = 3. *p<0.05.

To investigate the role of G6PD on the inflammatory response mediated by TNF-α stimulation, G6PD-kd A549 cells were established. As shown in S1A and S1B Fig, G6PD activities and protein expression were decreased by approximately 90% in G6PD-kd A549 cells compared with scramble control cells. The effect of G6PD deficiency on the COX-2 expression transcriptional level upon TNF-α treatment was determined. G6PD-kd A549 cells displayed decreased COX-2 mRNA expression in a time-dependent manner, and COX-2 promoter activity was attenuated by G6PD silencing (Fig 1A and 1B). At the translational level, TNF-α enhanced the accumulation of COX-2 protein in a time-dependent manner with a maximal response within 3–6 h in A549 scramble control cells (Fig 1C), similar to previous reports [29]. A decreased pattern of COX-2 protein was observed in G6PD-kd cells (Fig 1C). In parallel with the increased expression of COX-2, TNF-α also induced a time-dependent increase of PGE_2_ synthesis, the downstream arachidonic acid metabolite product of COX-2. G6PD-kd A549 cells exhibited less PGE_2_ synthesis than that of scramble control cells (Fig 1D). Our results demonstrated that G6PD acts as a positive regulator in the TNF-α-triggered inflammatory response and is not a result of the levels of TNFR1 expression in these two types of cells (S1B Fig).

Decreased COX-2 expression makes airway epithelial cells susceptible to viral infection [30, 31]. Using plaque assay, we found that the progeny viral particle derived from infected G6PD-kd cells was significantly higher compared with infected scramble control cells and the amount of viral particles was comparable with viral *N* gene expression (Fig 1E). These findings are consistent with our previous findings [26, 32]. We further analyzed whether impaired COX-2/PGE_2_ expression via TNF-α stimulation plays a role in increasing viral replication in G6PD-kd cells and used viral gene expression to represent viral replication. In doing so, we found that TNF-α inhibited viral replication in both scramble control and G6PD-kd cells (Fig 1F). Inhibition of TNF-α-induced COX-2 expression by celecoxib (COX-2 inhibitor) enhanced viral replication in scramble control cells but not in G6PD-kd A549 cells. These results indicate that TNF-α-induced COX-2 expression inhibits viral replication; increased susceptibility to viral infection in G6PD-kd A549 cells may occur by an impaired inflammatory response upon cytokine stimulation.

### Phosphorylation levels of MAPKs signaling are decreased in G6PD-kd cells upon TNF-α treatment

**Fig 2:**
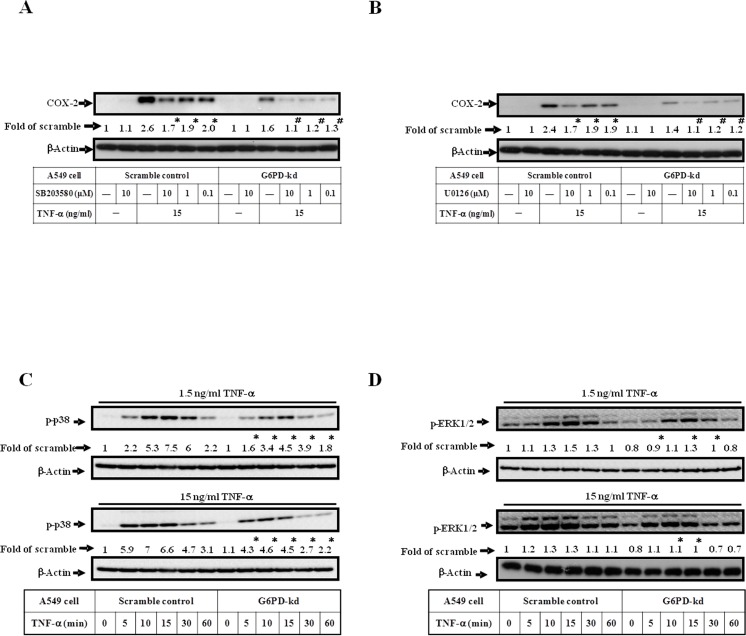
G6PD knockdown impairs the phosphorylation of MAPKs signaling. (A, B) The expression level of COX-2 was assessed by western blot in scramble control and G6PD-kd A549 cells treated with 15 ng/ml TNF-α or combined with MAPK inhibitor (SB203580, p38 inhibitor; U0126, MEK1/2 inhibitor) pre-treatment for 3 h. β-Actin expression was shown as the loading control. Numbers represent relative fold differences of protein levels on the basis of densitometer quantitation. Data are means ±SD of three separate experiments, *^,#^p<0.05 indicate significant difference between cells with or without inhibitor pretreatment upon TNF-α stimulation. (C, D) The phosphorylation level of p38 (C), ERK1/2 (D) were determined by western blot in scramble control and G6PD-kd A549 cells stimulated with 1.5 or 15 ng/ml TNF-α in different time courses. β-Actin expression was shown as the loading control. Numbers represent relative fold differences of protein levels on the basis of densitometer quantitation. Data are means ±SD of three separate experiments, *p<0.05 indicates significant difference comparing scramble control with TNF-α treatment at the corresponding time points.

TNF-α induces MAPKs activation in human airway epithelial cells, and MAPKs phosphorylation results in COX-2 induction [33]. Scramble control and G6PD-kd A549 cells were pretreated with SB203580 (an inhibitor of p38 MAPK) or U0126 (an inhibitor of MEK1/2) followed by TNF-α treatment, showing that the inhibition of the MAPKs pathway can impair TNF-α-induced COX-2 expression (Fig 2A and 2B). G6PD-kd cells displayed lower phosphorylation level of p38 and ERK1/2 than that of scramble control cells at various time points upon TNF-α treatment (Fig 2C and 2D). The JNK phosphorylation level was not altered (data not shown). These data indicate that decreased expression levels of COX-2 are tightly correlated with impaired phosphorylation levels of MAPKs signaling upon TNF-α treatment in G6PD-kd A549 cells when compared to scramble control cells.

### A decreased phosphorylation levels of c-JUN and NF-κB are observed in G6PD-kd A549 cells upon TNF-α treatment

**Fig 3:**
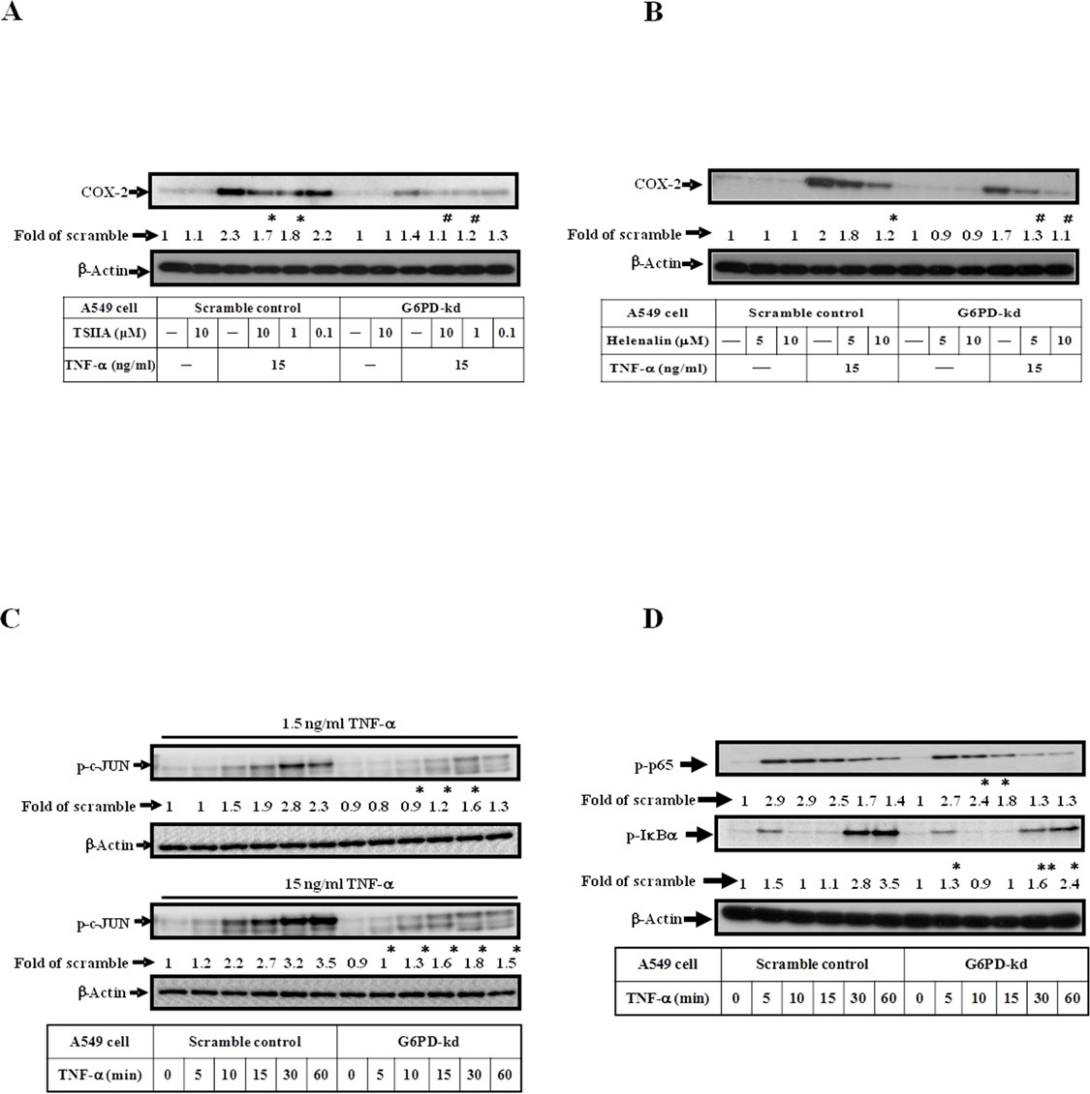
G6PD knockdown dysregulates the activation of c-JUN and NF-κB signaling. (A) Scramble control and G6PD-kd A549 cells were pretreated with Tanshinone IIA (TSIIA), AP-1 inhibitor for 2 h and then treated with 15 ng/ml TNF-α for 3 h. The expression level of COX-2 was assessed by western blotting assay. β-Actin expression was shown as the loading control. Numbers represent relative fold differences of protein levels on the basis of densitometer quantitation. Data are means ±SD of three separate experiments, *^,#^p<0.05 indicate significant difference between cells with or without inhibitor pretreatment upon TNF-α stimulation. (B) The expression level of COX-2 was determined by western blot under 15 ng/ml TNF-α stimulation or combined with pre-treatment of NF-κB inhibitor, Helenalin, for 3 h in scramble control and G6PD-kd A549 cells. β-Actin expression was shown as the loading control. Numbers represent relative fold differences of protein levels on the basis of densitometer quantitation. Data are means ±SD of three separate experiments, *^,#^p<0.05 indicate significant difference between cells with or without inhibitor pretreatment upon TNF-α stimulation. (C) The phosphorylation level of c-JUN was determined by western blot in scramble control and G6PD-kd A549 cells stimulated with 1.5 or 15 ng/ml TNF-α in different time courses. β-Actin expression was shown as the loading control. Numbers represent relative fold differences of protein levels on the basis of densitometer quantitation. Data are means ±SD of three separate experiments, *p<0.05 indicates significant difference between scramble control and G6PD-kd cells upon TNF-α treatment at the corresponding time points. (D) The phosphorylation levels of p65 and IκBα were investigated in scramble control and G6PD-kd A549 cells upon TNF-α treatment (15 ng/ml) in different time courses. β-Actin expression was shown as the loading control. Numbers represent relative fold differences of protein levels on the basis of densitometer quantitation. Data are means ±SD of three separate experiments, *p<0.05 and **p<0.01 represent levels of significant difference between scramble control and G6PD-kd cells upon TNF-α treatment at the corresponding time points.

Pretreatment with Tanshinone ΙΙA, an inhibitor of AP-1, downstream of the MAPKs pathway blocked TNF-α-induced COX-2 expression in scramble control and G6PD-kd A549 cells (Fig 3A). G6PD-kd A549 cells have a decreased phosphorylation level of c-JUN (Fig 3C). Inhibition of NF-κB activation by Helenalin attenuated TNF-α-induced COX-2 protein expression (Fig 3B). Decreased phosphorylation levels of p65 and IκBα were observed in G6PD-kd A549 cells upon TNF-α treatment (Fig 3D) compared with scramble control cells. These results indicate that impaired COX-2 expression induced by TNF-α treatment is not only correlated with decreased phosphorylation of MAPKs but also correlated with the reduced activation of NF-κB in G6PD-kd A549 cells.

### NOX is involved in the activation of TNF-α-mediated p38 MAPK phosphorylation and COX-2 expression

**Fig 4:**
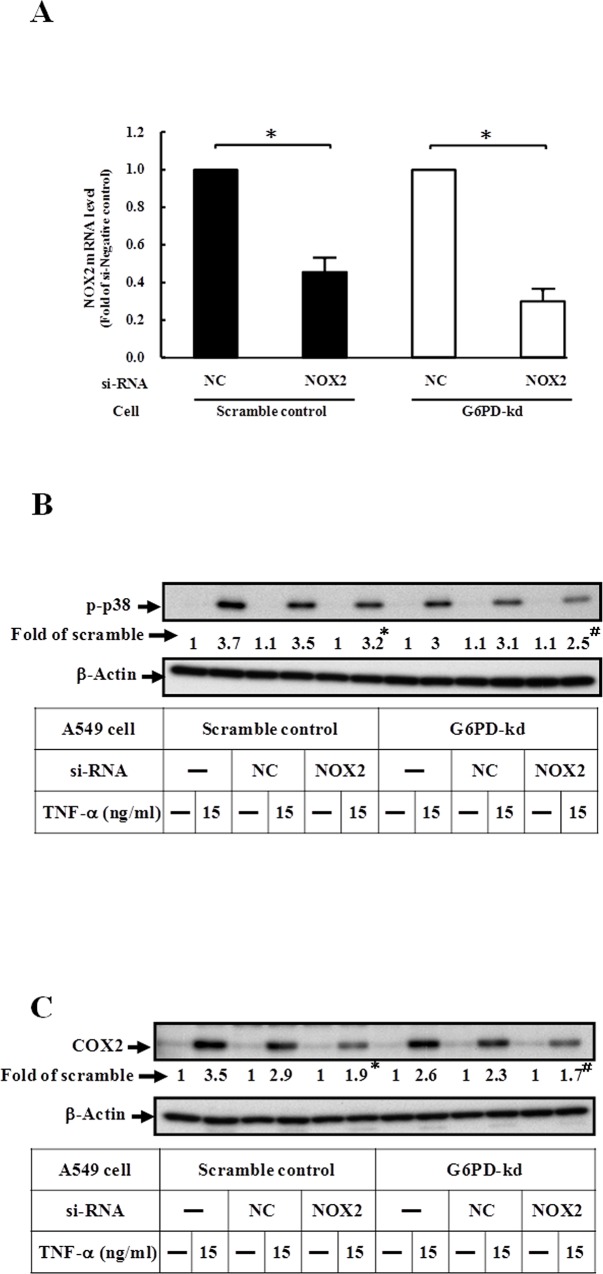
TNF-α-triggered p38 MAPK activation and COX-2 expression are mediated by NOX signaling. (A) The mRNA level of NOX2 was determined in universal negative control (NC) or NOX2-targeting siRNA transfected scramble control and G6PD-kd A549 cells. After transient transfection, cells were harvested for analyzing NOX2 mRNA expression. Data are the means ±SD, n = 3. *p<0.05 indicates significant difference between cells with or without NOX2 siRNA pretreatment. (B) The phosphorylation level of p38 was determined in scramble control and G6PD-kd A549 cells upon 15 ng/ml TNF-α stimulation combined with pre-treatment of universal negative control (NC) or NOX2-targeting siRNA. β-Actin expression was shown as the loading control. Numbers represent relative fold differences of protein levels on the basis of densitometer quantitation. Data are means ±SD of three separate experiments, *^,#^p<0.05 indicate significant difference between cells with or without NOX2 siRNA pretreatment upon TNF-α stimulation. (C) The expression level of COX-2 was determined under 15 ng/ml TNF-α stimulation for 3 h in universal negative control (NC) or NOX2-targeting siRNA transfected scramble control and G6PD-kd A549 cells. β-Actin expression was shown as the loading control. Numbers represent the relative fold differences of protein levels on the basis of densitometer quantitation. Data are means ±SD of three separate experiments, *^,#^p<0.05 indicate significant difference between cells with or without NOX2 siRNA pretreatment upon TNF-α stimulation.

The production of ROS by NOX is critical for cellular signaling and antimicrobial host defense [34–36]. In this study, we showed that transfection with NOX2 targeting siRNA reduced the NOX2 mRNA level (Fig 4A), and then attenuated TNF-α-induced p38 MAPK phosphorylation and COX-2 expression (Fig 4B and 4C) in both scramble control and G6PD-kd A549 cells; NF-κB was also inhibited (data not shown). To further confirm these results, a pharmacological inhibitor of NOX, DPI was used. As shown in S2A and S2B Fig, pretreated with DPI decreased the expression level of phospho-p38 and COX-2. These results revealed that TNF-α-triggered MAPK/NF-κB/COX-2 signaling is through NOX activation.

### G6PD is required for the up-regulation of NOX upon TNF-α treatment to combat against viral replication

**Fig 5:**
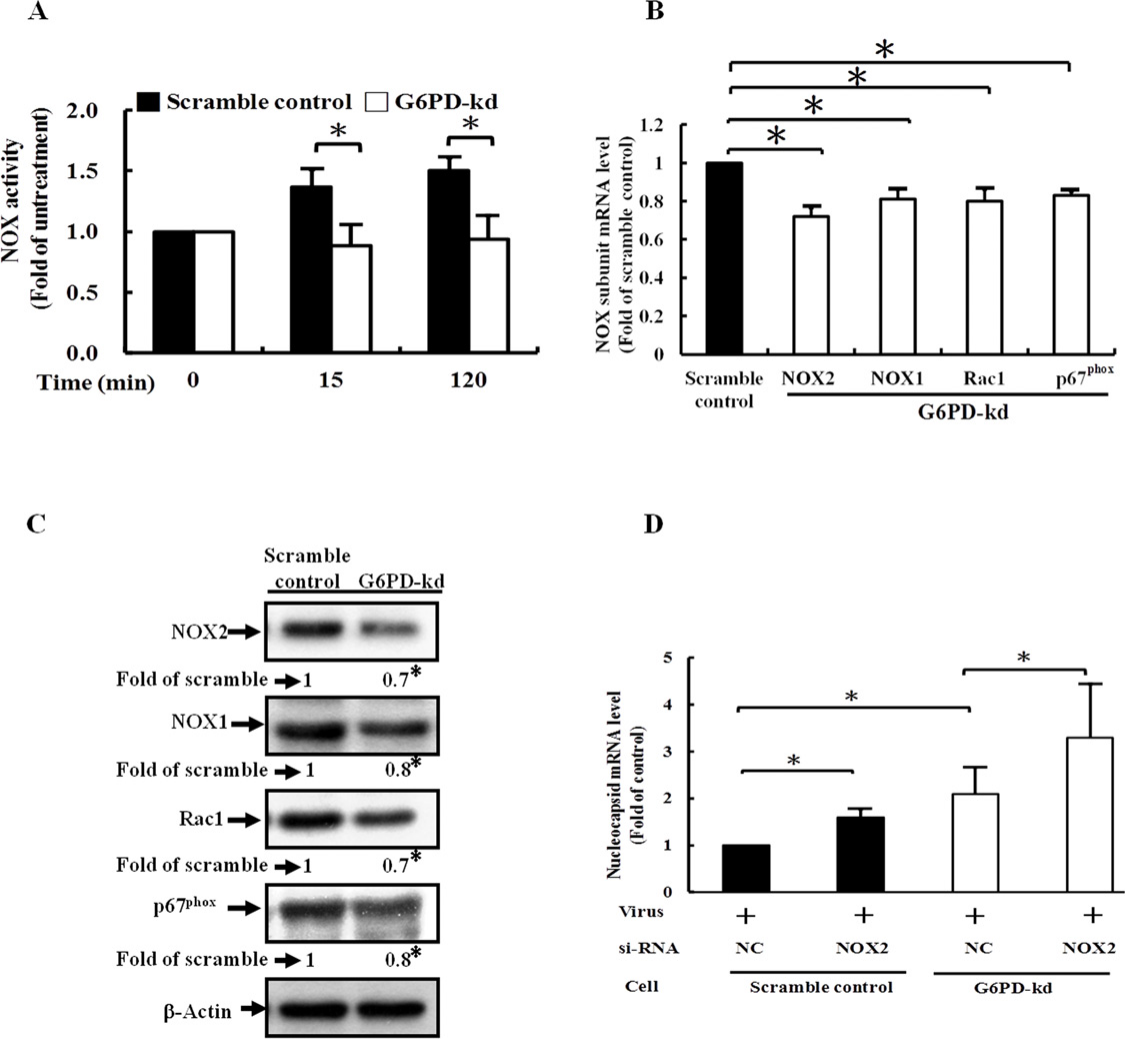
G6PD is required for the TNF-α-induced activation of NOX signaling and antiviral response. (A) The activity of NOX was measured by a lucigenin chemiluminescence assay. Data are the means ±SD, n = 3. *p<0.05. (B) The mRNA level of NOX subunits were determined by quantitative PCR in scramble control and G6PD-kd A549 cells. Data are the means ±SD, n = 3. *p<0.05. (C) The expression level of NOX subunits were determined by western blot in scramble control and G6PD-kd A549 cells. β-Actin expression was shown as the loading control. Numbers represent relative fold differences of protein levels on the basis of densitometer quantitation. Data are means ±SD of three separate experiments, *p<0.05 vs. scramble control. (D) Scramble control and G6PD-kd A549 cells were infected with coronavirus (0.1 MOI) for 8 h upon universal negative control (NC) or NOX2-targeting siRNA pretreatment, and the infected cells were harvested for analyzing viral mRNA expression. Data are the means ±SD, n = 3. *p<0.05.

G6PD provides NADPH for the activation of NOX [25]. A lucigenin chemiluminescence assay was used to ascertain the link between the G6PD knockdown and NOX activation. NOX activation was not significant in G6PD-kd A549 cells, while an increase in NOX activation was observed within 120 min in scramble control A549 cells upon TNF-α treatment (Fig 5A). In addition, G6PD knockdown decreased the expressions of NOX subunits, as indicated by the transcriptional and translational levels of the NOX subunits compared to those of the scramble control (Fig 5B and 5C). Moreover, NOX2 inhibition was accompanied by an increase in viral replication in scramble control and G6PD-kd A549 cells (Fig 5D and S2C Fig). These results suggest that G6PD modulated NOX activity by providing a substrate for NOX and affecting the expression of NOX subunits. In summary, these findings indicate that G6PD knockdown impairs TNF-α/MAPK/NF-κB/COX-2 signaling as a result of insufficient NOX activation, leading to the eventual increase in susceptibility to viral infection.

## Discussion

G6PD deficiency is linked to certain chronic and infectious diseases [1, 37–39]. The significance of the G6PD expression in the inflammatory response, especially in airway epithelial cells, is largely unknown. The present study is the first to show that G6PD knockdown inhibits MAPKs and NF-κB signaling upon TNF-α treatment, resulting in a significant decrease in COX-2 expression and PGE_2_ production. The underlying mechanism is a result of the decreased NOX activation as well as NOX subunits expression in G6PD-kd cells. These results suggest that G6PD deficiency impairs the cellular inflammatory response and has implications in the pathogenesis of infectious diseases in G6PD-deficient individuals.

G6PD derived NADPH is essential for NOX activation in vitro and in vivo [40–42]. G6PD not only provides a key substrate source for NOX, but may regulate the expression of NOX subunits because the mRNA levels of NOX2 components (p40^phox^, p47^phox^, and p67^phox^) are increased by G6PD overexpression in macrophages [8]. Consistent with this report, we have observed that G6PD knockdown markedly decreased the expression level of NOX subunits in parallel with an absence of NOX induction by TNF-α. G6PD can control nuclear NADPH-dependent superoxide production by NOX4 [43], and G6PD is colocalized with NOX in high glucose conditions, while providing NADPH for NOX activation [44] is an additional evidence to support the hypothesis that G6PD can modulate NOX activity.

Physiologically, the activation of NOX can trigger the production of chemokines and inflammation-associated proteins upon viral infection [19, 45, 46]. NOX2 and Duox play a role in the clearance of viral infection [35, 47, 48]. In the present study, we have found that the replication level of coronavirus is decreased by NOX inhibition and COX-2 activation as well as PGE_2_ secretion upon TNF-α treatment are diminished in G6PD-kd A549 cells. More interestingly, increased viral replication is observed by using COX-2 inhibitor in A549 cells. Together, these findings indicate that COX-2/PGE_2_ production actually improves anti-coronaviral infection in airway epithelial cells. Such notion is also supported by the finding that viral *N* gene is decreased by pretreatment with exogenous PGE_2_ (data not shown). These data are also in agreement with other group's findings, showing that COX-2/PGE_2_ production inhibits viral replication via the upregulation of antiviral cytokines (i.e. IL-32, IL-27, IFN-λ1) [30–31, 49] in A549 cells. In contrast to our observation, it has been reported that inhibition of COX-2 and PGE_2_ enhanced antiviral activity against virus infection in various kinds of cells [50–52]. Possible explanations for these contradicting results include different virus species, conditions for the pretreatment of TNF-α in cells, and different regulatory signalings between coronavirus and other upper respiratory tract virus for triggering innate immune response. Nevertheless, our new findings provide additional argument for the notion that NOX-mediated COX-2 signaling can be modulated by G6PD for triggering antiviral response.

Our new findings provide a link between G6PD and downstream NF-κB signaling that has been documented to be involved in several cellular responses to stimuli [53], particularly in the regulation of the immune response to pathogen stimulation. Dysregulation of NF-κB has been implicated in inflammation, viral infection, autoimmune diseases and improper immune development [54–56]. It has been found that G6PD-overexpressing macrophages increase the expression of proinflammatory genes via the activation of NF-κB [8]. Interestingly, we have also demonstrated that G6PD knockdown impairs NF-κB signaling triggered by NOX activity in the present report. When cells are infected by pathogens, cytokines are released, followed by the induction of inflammatory mediators. The increased levels of inflammatory mediators play a protective role or initiate an irreversible immune response leading to cell death. Thus, the current study provides novel evidence to suggest that TNF-α-mediated NF-κB signaling is, in part, modulated by a close interaction between G6PD and NOX. Additional studies are needed to elucidate the detailed interaction between these two well-known enzymes.

**Fig 6:**
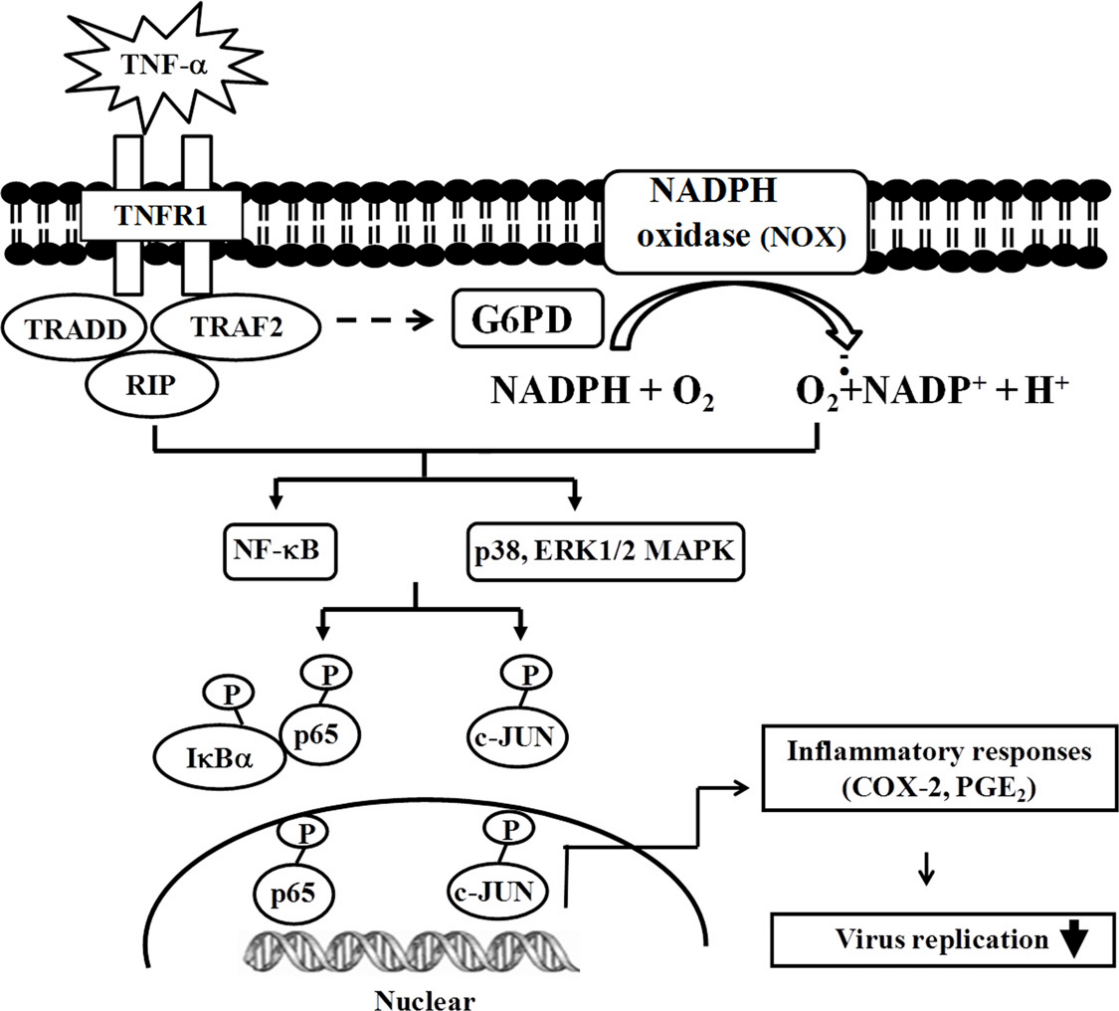
Proposed schematic representation of the signaling pathway involved in the TNF-α-induced NOX/MAPK/c-JUN/NF-κB/COX-2 signaling impaired by G6PD knockdown in A549 cells. Physiologically, stimulus by a low dosage of TNF-α causes cellular ROS production through the activation of NOX and downstream signaling (MAPK/NF-κB). The activated NOX/MAPK/c-JUN/NF-κB signaling is concomitant with the increased expression of COX-2 and production of PGE_2_. G6PD knockdown results in the reduced generation of cellular NADPH and an impairment of TNF-α-induced NOX activation. Consequently, COX-2 expression and PGE_2_ production are also less in G6PD-knockdown cells, suggesting the participation of G6PD in the TNF-α-induced inflammatory response against viral infection.

In conclusion, a mechanism is presented to explain how G6PD knockdown A549 cells can inhibit the TNF-α-mediated inflammatory response. TNF-α-mediated COX-2 expression is decreased by G6PD knockdown via the down-regulation of NOX-dependent pathways. G6PD knockdown can affect viral infection by decreasing the epithelial inflammatory response. A schematic representation depicts the signaling mechanism concerning G6PD deficiency on COX-2 expression (Fig 6). These findings enhance the understanding of how TNF-α signaling is affected by G6PD knockdown via a close interaction with NOX, and these novel findings should have potential clinical implications.

## Supporting Information

S1 Fig Establishment of G6PD-knockdown A549 cells. A549 cells were transfected with either G6PD-RNAi vector (G6PD-knockdown) or scrambled vector (Scramble control) by use of LF2000 as mentioned in Materials and Methods. The stably transfected cell lines were selected with 300 μg/ml G418. (A) Protein extracts of G6PD-knockdown A549 (G6PD-kd) and scramble control clones were used to measure G6PD activities. The results were presented as the mean values ± SD from three independent experiments. *p<0.05. (B) Equal amounts of proteins individually from the G6PD-kd and scramble control cells were applied to western blot analysis using G6PD and TNFR1 antibodies. β-Actin was present as the loading control. Numbers represent the relative fold differences of protein levels on the basis of densitometer quantitation. Data are means ±SD of three separate experiments, *p<0.05 vs. scramble control.
(TIF)

S2 Fig NOX is implicated in the regulation of TNF-α-mediated signaling and antiviral response. (A) The phosphorylation level of p38 was determined in scramble control (Upper) and G6PD-kd A549 cells (Lower) upon different dosages of TNF-α stimulation combined with or without pre-treatment of DPI for 10 min. Quantitations of p-p38 MAPK protein expression was obtained by densitometric analysis. Data are means ±SD of three separate experiments, *^,#^p<0.05 vs. cells upon TNF-α stimulation without DPI pretreatment. (B) The expression level of COX-2 was determined under TNF-α stimulation or combined with pre-treatment of DPI for 3 h in scramble control and G6PD-kd A549 cells. β-Actin expression was shown as the loading control. Numbers represent the relative fold differences of protein levels on the basis of densitometer quantitation. Data are means ±SD of three separate experiments, *^,#^p<0.05 vs. cells upon TNF-α stimulation without DPI pretreatment. (C) Scramble control A549 cells were infected with coronavirus (0.1 MOI) for 8 h upon 10 μM DPI pretreatment, and the infected cells were harvested for analyzing viral mRNA expression. Data are the means ±SD, n = 3. *p<0.05.
(TIF)
