# Supplementary figures and images for: Diminished COX-2/PGE2-Mediated Antiviral Response Due to Impaired NOX/MAPK Signaling in G6PD-Knockdown Lung Epithelial Cells
Source: PLoS One. 2016 Apr 20;11(4):e0153462. doi: 10.1371/journal.pone.0153462 (PMC4838297; doi:10.1371/journal.pone.0153462)

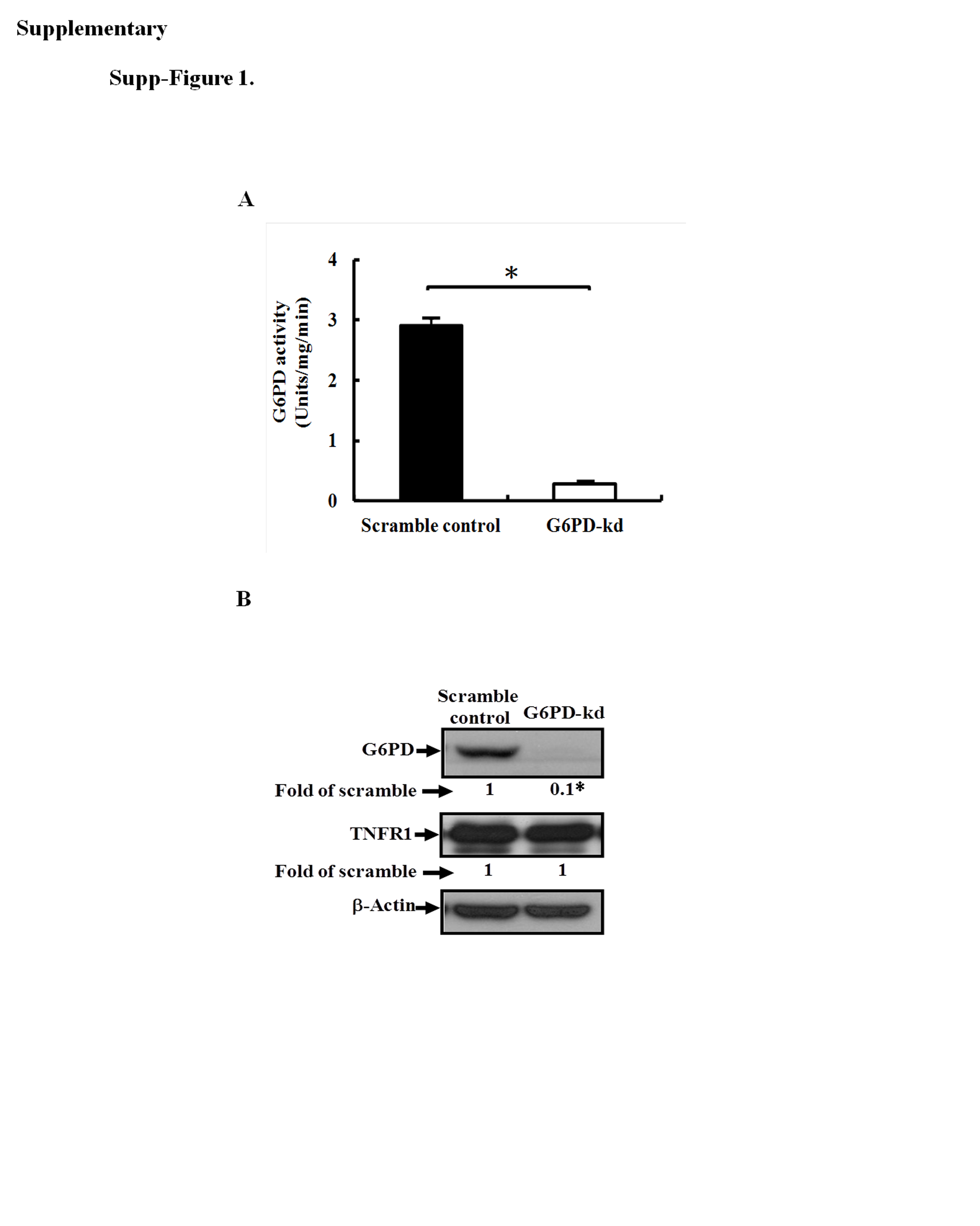

Supplement: S1 Fig — A549 cells were transfected with either G6PD-RNAi vector (G6PD-knockdown) or scrambled vector (Scramble control) by use of LF2000 as mentioned in Materials and Methods. The stably transfected cell lines were selected with 300 μg/ml G418. (A) Protein extracts of G6PD-knockdown A549 (G6PD-kd) and scramble control clones were used to measure G6PD activities. The results were presented as the mean values ± SD from three independent experiments. *p<0.05. (B) Equal amounts of proteins individually from the G6PD-kd and scramble control cells were applied to western blot analysis using G6PD and TNFR1 antibodies. β-Actin was present as the loading control. Numbers represent the relative fold differences of protein levels on the basis of densitometer quantitation. Data are means ±SD of three separate experiments, *p<0.05 vs. scramble control. (TIF) [file pone.0153462.s001.TIF]

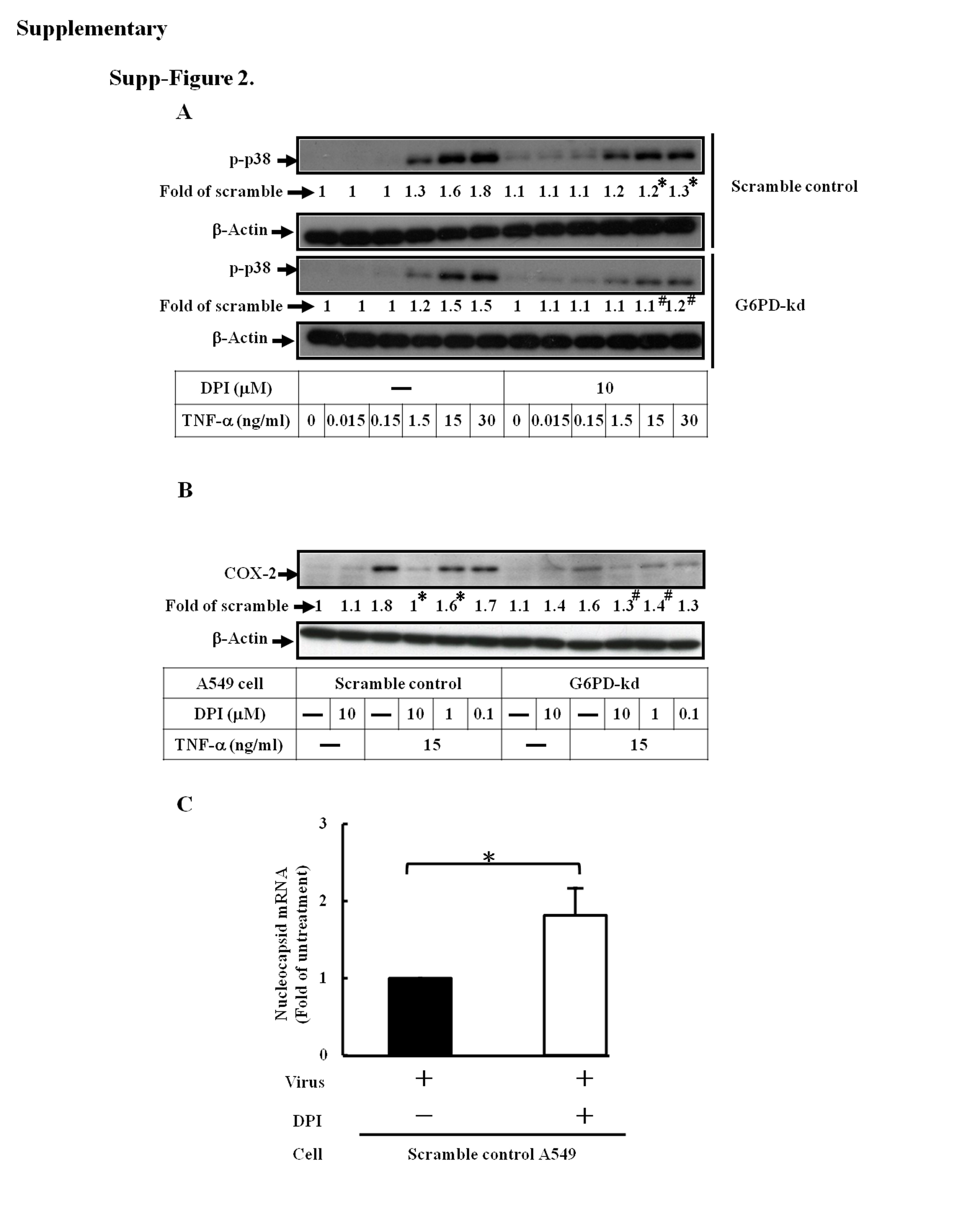

Supplement: S2 Fig — (A) The phosphorylation level of p38 was determined in scramble control (Upper) and G6PD-kd A549 cells (Lower) upon different dosages of TNF-α stimulation combined with or without pre-treatment of DPI for 10 min. Quantitations of p-p38 MAPK protein expression was obtained by densitometric analysis. Data are means ±SD of three separate experiments, *,#p<0.05 vs. cells upon TNF-α stimulation without DPI pretreatment. (B) The expression level of COX-2 was determined under TNF-α stimulation or combined with pre-treatment of DPI for 3 h in scramble control and G6PD-kd A549 cells. β-Actin expression was shown as the loading control. Numbers represent the relative fold differences of protein levels on the basis of densitometer quantitation. Data are means ±SD of three separate experiments, *,#p<0.05 vs. cells upon TNF-α stimulation without DPI pretreatment. (C) Scramble control A549 cells were infected with coronavirus (0.1 MOI) for 8 h upon 10 μM DPI pretreatment, and the infected cells were harvested for analyzing viral mRNA expression. Data are the means ±SD, n = 3. *p<0.05. (TIF) [file pone.0153462.s002.TIF]
